# Safety of second-line chemotherapy with non-conventional fotemustine schedule in recurrent high grade gliomas: a single institution experience

**DOI:** 10.1007/s11060-013-1147-x

**Published:** 2013-05-24

**Authors:** P. Gaviani, G. Simonetti, A. Salmaggi, E. Lamperti, A. Silvani

**Affiliations:** 1 Neuro-Oncology Department, Fondazione IRCCS Istituto Neurologico Carlo Besta, Milan, Italy; 2 SC Neurologia Ospedale Manzoni Lecco, Lecco, Italy

## Introduction and study objective

Fotemustine (FTM) is a third-generation nitrosourea. Recently, several groups have studied the use of FTM in high grade glioma (HGG) patients recurring after standard radiotherapy (RT) and temozolomide (TMZ) treatment [1–3]. With the FTM standard schedule (dose of 100 mg/sqm/week for 3 consecutive weeks as an induction treatment, followed by 100 mg/sqm every 3 weeks, after a 5-week rest, as maintenance treatment) myelosuppression represents the most common adverse event, mainly occurring during the induction phase of treatment. In a modified schedule proposed by Addeo et al. [4], FTM was administered at a dose of 80 mg/sqm every 2 weeks for five consecutive administrations as the induction phase and every 4 weeks at 80 mg/sqm as the maintenance phase. Accordingly, in a prospective single-institution study, we addressed the toxicity of a modified FTM schedule in patients with HGG at 1st and 2nd progression after failure of RT and TMZ.

## Methods and statistical analysis

Between January 2011 and October 2012, 97 consecutive patients with a diagnosis of HGG at first or second recurrence after standard treatment were treated with an outpatient regimen with a non conventional schedule of FT as proposed by Addeo et al. The protocol was approved by our Institutional Review Board, and each patient provided written informed consent before initiation of treatment. In this setting, clinical, radiological and laboratory data of all patients treated were collected. In particular complete blood count (CBC) with differential, biochemistry panel (BUN, creatinine, liver enzymes, glucose), erythrocyte sedimentation rate (VES), C-reactive protein (CRP) and CD4 count were assessed every cycle. Toxicity was evaluated according to the Common Terminology Criteria for Adverse Events (CTCAE; version 3.0). Median progression-free survival at 6 months (PFS-6) and median overall survival (m-OS) were calculated for the whole group of patients, as secondary study endpoints. Descriptive statistics were used to summarize relevant study information. PFS and OS were calculated by the Kaplan–Meier method.

## Results

Clinical characteristics and toxicity profile of the 97 patients (59 male and 38 female) included in the study are summarized in Table 1 and 2. Overall, patients received a total of 431 chemotherapy cycles: the median number of cycles was 5 (range, 2–13). Only one patient interrupted chemotherapy due to hematological grade IV toxicity. In all patients, CD4 + lymphocytes counts were monitored: 18.5 % of patients developed grade 3–4 CD4 + lymphopenia (<200 cell/μL). All patients with grade 3/4 CD4 + lymphopenia received prophylactic cotrimoxazol. None of these patients developed *Pneumocystis carinii* pneumonia. Moreover, no death was considered to be closely related to chemotherapy toxicity. Major non hematological toxicities (grade 3–4) concerned mainly hepatic enzymes, particularly GGT that increased in 5.5 % of cases Minor toxicities (grade I–II) were anemia (42.1 %), thrombocytopenia (15.1 %), leukopenia (9.2 %), AST/ALT increase (approximately 40 %), worsening of renal function (2.8 %), and gastrointestinal toxicity (6.2 %). Infections were observed in 9 cases (2 pulmonary, 2 urinary trait, 5 other) and deep venous thrombosis in 7 cases.

**Table 1:** Patient’s characteristics

Patients n = 97	
*Clinical characteristics*	
Median age, years (range)	56.5 (20–72)
Median KPS	80 (60–100)
Male/female	59/38
Histotype at diagnosis	
Glioblastoma multiforme	58
Grade III gliomas	21
Grade II gliomas	18
Surgery at recurrence	
Yes	39
No	58
*Chemotherapy treatment*	
Lines of chemotherapy	
Second line chemotherapy (number of patients)	83
Third line chemotherapy (number of patients)	14
Total cycles administered	431
Median cycles received, number (range)	5 (2–13)
Induction phase completed (number of patients)	20
All cycles completed (number of patients)	14
*Survival data for the whole group of patients*	
6 months -PFS	38, 2 %
6 months-OS	56 %
12 months -OS	18 %
Median PFS	16 weeks
Median OS	30 weeks

**Table 2:** Summary of chemotherapy related toxicity

Toxicity	Grade 1	Grade 2	Grade 3	Grade 4
Haematological				
Anemia	176 (40.8 %)	6 (1.3 %)	–	–
Thrombocytopenia	60 (13.9 %)	5 (1.2 %)	–	1 (0.3 %)
Leukopenia	29 (6.7 %)	11 (2.5 %)	–	1 (0.3 %)
CD4 + lymphopenia	23 (5.3 %)	98 (22.7 %)	79 (18.3 %)	1 (0.2 %)
*Non haematological*				
Hypertransaminasemia				
>AST	35 (8.1 %)	4 (0.9 %)	–	–
>ALT	107 (24.8 %)	22 (5.1 %)	5 (1.2 %)	–
>Gamma GT	89 (20.6 %)	36 (8.3 %)	18 (4.2 %)	6 (1.4 %)
Renal				
Creatinine	12 (2.8 %)	–	–	–
Gastrointestinal				
Nausea/vomiting	21 (6.2 %)	–	–	–

Other chemotherapy treatment related adverse event	Number of cases (%)
Infections	9 (9.2 %)
Pulmonary	2 (2 %)
Urinary trait	2 (2 %)
Other	5 (5.1 %)
Mycosis/parasitosis	12 (12.3 %)
Deep venous thrombosis	7 (7.2 %)
Fatal	2 (2 %)

## Discussion and conclusions

The management of patients with recurrent HGG is particularly challenging due to the lack of a standard of care. Significant evidence exists from preclinical and clinical studies for the activity of FTM particularly in recurrent malignant glioma. In the FTM standard regimen, hematological toxicity represents the most important side effect, with grade 3–4 toxicities in nearly 40 % of patients. Thus, to improve the safety of FTM use, several groups have reported experiences with alternative schedules [1, 2]; in particular Addeo et al. reported results of an alternative FTM schedule where the total FTM dose at the end of induction was maintained but with a different fractionation [4]. In 2008, our group reported the results of a combination schedule of FTM with procarbazine (PCB) in recurrent malignant glioma patients [5]. In that study, we used PCB at a dose of 450 mg/day on days 1–3 and FTM at a dose of 110 mg/sqm in a single monthly administration on day 3. A limitation of our combination schedule was most likely the abolition of the induction phase, consequently reducing the total amount of FTM administered. Thus, because we are now convinced of the crucial role of the induction phase in the FTM treatment, we used an alternative schedule in the present paper on recurrent glioma patients that would ensure an acceptable toxicity profile. The treatment was very well tolerated, the analysis on collected data showed a good safety profile with only few grade 3–4 toxicities. Only in one case, after three infusions of FTM, a young girl had severe hematological toxicities that required treatment discontinuation. Only two fatal pulmonary embolisms were recorded, although they were not closely correlated with chemotherapy but with the tumor itself.

In our series of patients the 6-PFS was 38.2 % (± 0.0695); the median OS was 30 weeks, with a OS-6 of 56 % and a OS-12 of 18 %. Even if this is a prospective study with a limited number of patients, and our patient population is heterogeneous (including grade III and grade IV gliomas) our results in term of efficacy are quite comparable as 6-PFS and responses to other studies on standard FT schedule; however at the moment whether it could be more effective than the conventional regimen remains unclear, but limited toxicity compared to conventional schedule makes it safe to use in clinical practice.

In conclusion, although the present study utilized a limited number of patients, it confirms the safety of the alternative FTM schedule proposed by Addeo et al.
